# French Prospective Clinical Evaluation of the Aptima Mycoplasma genitalium CE-IVD Assay and Macrolide Resistance Detection Using Three Distinct Assays

**DOI:** 10.1128/JCM.00579-17

**Published:** 2017-10-24

**Authors:** Chloé Le Roy, Sabine Pereyre, Nadège Hénin, Cécile Bébéar

**Affiliations:** a Univ. Bordeaux, USC EA 3671 Mycoplasmal and Chlamydial Infections in Humans, Bordeaux, France; b INRA, USC EA 3671 Mycoplasmal and Chlamydial Infections in Humans, Bordeaux, France; c CHU Bordeaux, Laboratoire de Bactériologie, Bordeaux, France; Marquette University

**Keywords:** Mycoplasma genitalium, transcription-mediated amplification, real-time PCR, clinical specimens, macrolide resistance

## Abstract

The aim of this study was to evaluate the clinical performance of the Aptima Mycoplasma genitalium transcription-mediated amplification (MG-TMA) CE-marked for *in vitro* diagnosis (CE-IVD) assay for the detection of Mycoplasma genitalium in male and female clinical samples in comparison with the in-house real-time PCR (in-house PCR) assay routinely used in our laboratory. A total of 1,431 clinical specimens obtained from 1,235 patients were prospectively collected at the Bacteriology Department of Bordeaux University Hospital (France). Additional research-use-only Aptima M. genitalium transcription-mediated amplification (TMA) assays, Alt1-TMA and Alt2-TMA, were performed on discordant specimens to determine M. genitalium infection status. All confirmed M. genitalium-positive specimens were tested for macrolide resistance using three assays: the in-house 23S rRNA FRET PCR assay, the SpeeDx ResistancePlus MG assay and the nested reverse transcription-PCR (RT-PCR) sequencing assay. The comparison of the MG-TMA assay with the in-house PCR results showed a moderate correlation (kappa value, 0.69). The MG-TMA assay had higher clinical sensitivity compared to that of the in-house PCR assay (100% versus 59.74%, respectively) and similar specificity (99.10% versus 100%, respectively) for M. genitalium detection. In this study, the prevalence of M. genitalium infection was 5.90% (72/1,220 patients). The nested RT-PCR sequencing assay was the most sensitive but the most laborious assay for detecting macrolide-resistance-associated mutations. The prevalence of resistance was 8.33% (6/72). To our knowledge, this is the first clinical evaluation of the MG-TMA CE-IVD assay. The MG-TMA assay performed on the automated Panther system is a very sensitive and specific method for the detection of M. genitalium in clinical specimens.

## INTRODUCTION

Mycoplasma genitalium is an important sexually transmitted organism involved in nongonococcal urethritis in men and is associated with female cervicitis and pelvic inflammatory disease in women (1–3). Nucleic acid amplification tests (NAATs) that detect M. genitalium-specific nucleic acids in clinical specimens are the only available methods for diagnosis, due to the difficulties in isolating M. genitalium by culture (3). Unfortunately, no FDA-approved commercial assays for the detection of M. genitalium are available yet. However, several NAATs based on real-time PCR or transcription-mediated amplification (TMA) have been described for the molecular detection of M. genitalium (3–9). Several companies have commercialized CE-marked multiplex PCR assays for the detection of sexually transmitted pathogens, including M. genitalium (6, 7, 9, 10). As an alternative to PCR, Hologic, Inc. developed a research-use-only (RUO) TMA assay for the detection of M. genitalium (8, 11, 12) and recently commercialized the Aptima Mycoplasma genitalium TMA (MG-TMA) assay CE-marked for *in vitro* diagnosis (CE-IVD), which is available on the Panther platform. Commercial tests that have been CE-marked for document conformity usually suffer from limited clinical validation. Consequently, it is very important that diagnostic laboratories assess the clinical utility of these commercial assays.

Only a few antimicrobial classes have activity against mycoplasmas and ureaplasmas, including tetracyclines, macrolides, and fluoroquinolones (13). M. genitalium has demonstrated a remarkable capability of developing resistance to almost all antimicrobials used to date, including macrolides and fluoroquinolones. With the increasing prevalence of resistant strains in Europe, the 2016 European guideline on M. genitalium infections has recommended completing the molecular detection of M. genitalium with an assay capable of detecting macrolide-resistance-associated mutations (14). A variety of methods are available for this purpose (9, 15–20).

In the present study, we aimed to prospectively evaluate the clinical performances of the MG-TMA CE-IVD assay (Hologic) for the detection of M. genitalium in male and female clinical specimens at the Bordeaux University Hospital in comparison with the in-house real-time PCR (in-house PCR) assay routinely used in our laboratory. We also assessed the macrolide-resistance-associated 23S rRNA mutations from M. genitalium-positive specimens using three distinct assays.

## RESULTS

### Performance of Aptima Mycoplasma genitalium assay in clinical specimens.

A total of 1,431 specimens were evaluated using the MG-TMA assay and the results were compared to those obtained with the in-house PCR assay routinely used for the detection of M. genitalium (Table 1). No inhibition of amplification of the internal control included in the in-house PCR was observed with specimens collected in Aptima medium, validating the use of the DNA extraction and in-house PCR performed in Aptima transport medium as a comparative assay. A total of 1.46% (21/1,431) results were considered invalid using the MG-TMA assay. Eighteen samples (13 vaginal swabs, one endocervical swab, one Douglas/peritoneal fluid sample, one rectal swab, one urethral swab, and one urine sample) were considered invalid due to insufficient sample volume after using the Aptima Combo 2 assay for Chlamydia trachomatis and Neisseria gonorrhoeae detection. Three vaginal swabs (3/1,431; 0.21%) were considered invalid because the internal control was not amplified. The latter three samples were negative for C. trachomatis and N. gonorrhoeae using the Aptima Combo 2 assay, which does not include an internal control. Consequently, comparative analysis was eventually performed on 1,410 specimens (1,431 minus the 21 invalid specimens) with valid results for both M. genitalium assays.

**TABLE 1:** Comparison of the M. genitalium detection results obtained with the MG-TMA assay and the in-house PCR assay

MG-TMA assay result	In-house PCR result		Overall % agreement (CI 95%)^*a*^	Kappa statistic
	Positive	Negative		
Positive	46	38	97.30 (96.32–98.03)	0.69
Negative	0	1326		
Invalid^*b*^	2	19		

a CI, confidence interval.

b 18 specimens gave invalid results by MG-TMA assay due to insufficient specimen volume; 3 specimens gave invalid results due to lack of internal control amplification.

The comparison of the MG-TMA assay and the in-house PCR showed a moderate correlation, with a kappa value of only 0.69 and an overall agreement of 97.30% (Table 1). The positivity rate was significantly higher with the MG-TMA assay (5.95%; 84/1,410) compared to with the in-house PCR assay (3.2%; 46/1,410) (*P* < 0.001; McNemar's test).

A percentage of the samples (2.69%; 38/1,410) gave discordant results, with a positive signal in only the MG-TMA assay (Table 1). Of the 38 specimens, 31 were from women (23 vaginal swabs, six endocervical swabs, one pharyngeal swab, and one urine sample) and seven were from men (two urine samples, two urethral swabs, two rectal swabs, and one pharyngeal swab). The 38 discordant specimens mixed with 38 randomly selected concordant M. genitalium-negative specimens were blindly tested with two alternate Aptima RUO TMA assays, Alt1-TMA and Alt2-TMA. The M. genitalium infection status was determined as described below. Of the 38 concordant M. genitalium-negative specimens, 36 were confirmed negative using both Alt-1 and Alt-2 TMA assays, one was reported positive by the Alt1-TMA assay only and one was reported positive by the Alt2-TMA assay only. The M. genitalium statuses of all the 38 concordant M. genitalium-negative specimens were thus confirmed as M. genitalium negative. Of the 38 discordant specimens, 26 were confirmed positive for M. genitalium, whereas 12 were confirmed to be M. genitalium negative. Finally, of the 1,410 specimens enrolled with valid results, a total of 72 specimens were confirmed positive for M. genitalium (5.1%). The rates of positivity were 5.3% (67/1273), 6.7% (2/30), 7.1% (3/42), 0 (0/42), and 0 (0/23) for genital specimens, pharyngeal swabs, rectal swabs, sperm specimens, and other types of specimens, respectively.

The comparison of the MG-TMA assay results with M. genitalium infection status showed a very good correlation, with a kappa value of 0.93 and a percent agreement of 99.15% (95% confidence interval [CI], 98.52 to 99.51). In contrast, the comparison of the in-house PCR results with M. genitalium infection status showed a lower correlation, with a kappa value of 0.73. The MG-TMA assay had a clinical sensitivity of 100% and a clinical specificity of 99.10% for M. genitalium detection, with a positive predictive value of 85.71% (Table 2). In comparison, the in-house PCR assay had a clinical sensitivity of 59.74% and a clinical specificity of 100% for M. genitalium detection, with a positive predictive value of 100%.

**TABLE 2:** Clinical sensitivity and specificity for M. genitalium detection based on M. genitalium infection status^*a*^

Assay	Assay result	Mg infection status^*b*^			Sensitivity (% [95% CI])	Specificity (% [95% CI])	PPV (% [95% CI])	NPV (% [95% CI])
		Positive	Negative	Total				
In-house PCR	Positive	46	0	46				
	Negative	31	1,333	1,364	59.74 (48.58–69.98)	100 (99.71–100)	100 (92.29–100)	97.73 (96.79–98.39)
	Any	77	1,333	1,410				
MG-TMA	Positive	72	12	84				
	Negative	0	1,326	1,326	100 (94.93–100)	99.10 (98.44–99.49)	85.71 (76.67–91.63)	100 (99.71–100)
	Any	72	1,338	1,410				

a CI, confidence interval; Mg, M. genitalium; NPV, negative predictive value; PPV, positive predictive value.

b Consensus results for M. genitalium detection represent positivity for two of three tests (not including the test being evaluated) and were used as the reference standard to compare test performances.

Overall, of the 1,235 patients enrolled in the study, the M. genitalium infection status could be determined for 1,220 patients. The M. genitalium infection prevalence was 5.65% (69/1,220) with 5.67% (59/1,040) of infections in women and 5.55% (10/180) in men.

### Prevalence of M. genitalium macrolide resistance.

The 72 specimens with a confirmed M. genitalium-positive infection status were tested for macrolide-resistance-associated mutations using three distinct assays: (i) the in-house 23S rRNA fluorescence resonance energy transfer PCR (FRET PCR) assay (17), (ii) the ResistancePlus MG assay (9, 20), and (iii) the nested RT-PCR and Sanger sequencing assay of M. genitalium 23S rRNA, used as reference standard (11). The 23S rRNA nested RT-PCR sequencing assay presented the highest sensitivity (100%) to amplify M. genitalium DNA/RNA compared to the ResistancePlus MG assay (63.89%) and the FRET PCR assay (52.78%) (Table 3). Among the 6 specimens harboring M. genitalium macrolide-resistance-associated mutations, 3 specimens were detected by the FRET PCR and 4 were detected by the ResistancePlus MG assay (Table 3; see also Table S2 in the supplemental material). Two specimens were detected as mutated by the ResistancePlus MG assay but were identified as wild type by the two other assays. Sequencing chromatograms were carefully examined and no double peaks were observed at the positions targeted by the ResistancePlus MG assay, A2058 or A2059, suggesting the absence of a mixture of mutant and wild-type M. genitalium. Thus, the macrolide resistance status was confirmed as wild type for these two specimens. Overall, 6 specimens harbored a 23S rRNA macrolide-resistance-associated mutation, 4 specimens harbored an A2059G substitution, and 2 specimens harbored an A2059C mutation. The prevalence of macrolide resistance was 8.33% (6/72) in the M. genitalium-positive population. Of the six patients with mutated strains, one harboring an A2059G substitution had received an extended azithromycin regimen (1.5 g for 5 days) 4 months earlier for a M. genitalium infection. The M. genitalium infection was then unsuccessfully treated with doxycycline for 2 weeks. Previous treatments and therapeutic outcomes were not available for the other five patients.

**TABLE 3:** Detection of macrolide resistance in the 72 M. genitalium-positive specimens^*a*^

Macrolide resistance assay	No. of specimens yielding Mg DNA/RNA amplification		Sensitivity (% [no./total of Mg DNA/RNA amplification]) (95% CI)	23S rRNA mutation		
	Successful	Unsuccessful		Absent	Present	No. of specimens with accurate macrolide resistance
23S rRNA FRET PCR	38	34	52.78 (38/72) (41.40–63.87)	35	3	3
ResistancePlus MG	46	26	63.89 (46/72) (52.35–74.02)	40	6	4
RT-PCR and sequencing	72	0	100 (72/72) (94.93–100)	66	6	6

a Mg, M. genitalium; CI, confidence interval.

## DISCUSSION

This study is the first evaluation published to date of the MG-TMA CE-IVD assay for the fully automated Panther platform. The study involved both symptomatic and asymptomatic men and women and all types of specimens for which M. genitalium detection was prescribed. The MG-TMA assay had a much higher sensitivity and a similar specificity compared to the in-house PCR assay for M. genitalium detection. Higher sensitivity and similar specificity of TMA compared to real-time PCR was previously reported for M. genitalium detection (8). The positive predictive values (PPVs) of the two tests were significantly different, as the confidence intervals did not overlap (Table 2); the PPV of 85.71% for the MG-TMA assay versus 100% for the in-house PCR assay indicates a risk of yielding more false M. genitalium-positive results with the MG-TMA assay. The necessity of confirming initial positive results could be of importance, at least for low prevalence populations. Considering that TMA assays have a higher sensitivity than real-time PCR assays, the confirmatory test should also be a TMA assay. The commercialization of an alternative TMA assay, such as Alt1-TMA or Alt2-TMA that targets a distinct region of the M. genitalium genome, may be useful.

In the present study that included 85% female patients, the prevalence of M. genitalium infection was 5.65% in 2016 at the Bordeaux University Hospital, a percentage that is slightly higher than the 3.40% previously reported in a multicentric prevalence study from individuals screened for sexually transmitted infections in French University Hospitals from 2014 to 2015 using a commercial real-time PCR assay (21). This difference in prevalence may not be associated with the male/female proportion as females were predominant in both studies. The difference in prevalence may rather be associated with the difference in sensitivity between TMA and real-time PCR, highlighting the importance in the choice of the M. genitalium detection method. In addition, the observed 5.65% M. genitalium prevalence makes M. genitalium the second most prevalent sexually transmitted microorganism after C. trachomatis (prevalence of 6.2% in this study, data not shown), as was previously reported in other European studies (21, 22).

Macrolide antibiotics are the first-line treatment for M. genitalium infections; however, macrolide resistance rates of up to 40% have been observed in several countries (11, 14, 15, 23). The 2016 European guideline on M. genitalium infection recommends that a molecular diagnosis should be followed with an assay for macrolide resistance (14). Unlike N. gonorrhoeae, M. genitalium is extremely difficult to culture, greatly limiting the option for culture-based antibiotic susceptibility testing. As a consequence, molecular resistance tests have recently been developed (9, 16–19). In this study, we evaluated three methods, two in-house (11, 17) and one commercial assay (20), to determine the presence of macrolide resistance-associated mutations. We observed that the MagNA Pure 96 automated extraction system, used for DNA extraction from clinical specimens in Aptima transport medium, can be used to perform PCR detection of macrolide resistance-associated mutations. Indeed, no increase in the cycle threshold was obtained for the amplification of the in-house PCR internal control, and no invalid results were obtained using the ResistancePlus MG assay, which also includes an internal control. The 23S rRNA nested RT-PCR sequencing assay presented the highest sensitivity to amplify M. genitalium DNA/RNA compared to the ResistancePlus MG assay and to the FRET PCR assay. It was the only method that gave a result for all the M. genitalium-positive specimens. However, it was the most laborious and time-consuming method, and it is not adapted for routine use compared to the two other assays. Our assessment of macrolide antibiotic resistance reported the detection of the common A2059G substitution, known to result in a high-level macrolide resistance phenotype (22), and the A2059C mutation, which is less common (19, 24, 25). Surprisingly, in this study, the prevalence of macrolide resistance-associated mutations among the M. genitalium-positive patients was only 8.33%, a percentage significantly lower than the prevalence of 17.2% described for M. genitalium at the Bordeaux University Hospital from 2013 to 2014 (26). As multiple assays were used to detect macrolide resistance-associated mutations, the accuracy of the results was extremely unlikely to be the issue. It should be noted that in the present study, there were no patients from sexually transmitted disease (STI) clinics, which was not the case in the previous study. Thus, the population studied here is likely to be a lower risk population who may have been exposed to less azithromycin treatments for STIs.

In conclusion, the MG-TMA assay is a simple and accurate method for the detection of M. genitalium in clinical specimens; it offers very sensitive diagnostic and flexible throughput on a fully automated all-in-one platform. The assay allows prompt results, validated by the use of an internal control. Because macrolide resistance is an increasing problem throughout the world, further efforts should be made to combine M. genitalium TMA detection with simple and highly sensitive methods of detecting macrolide resistance-associated mutations to fulfil the new European guidelines on M. genitalium infection and to optimize treatment regimens.

## MATERIALS AND METHODS

### Study population.

Between February and June 2016, clinical specimens from 1,235 patients were prospectively collected at the Bacteriology Department of Pellegrin Hospital, Bordeaux University Hospital, France, from 1,053 women and 182 men who were hospitalized at the Bordeaux University Hospital or were consulting at the family planning center or at the abortion and reproductive biology departments of the Bordeaux University Hospital. No STI clinic patients were included in this study. Approximately two-thirds of patients were asymptomatic and one-third were symptomatic.

### Specimen collection.

Overall, 1,431 specimens, for which testing for M. genitalium was prescribed, were systematically and prospectively collected. The specimen types included 749 vaginal swabs, 294 endocervical swabs, 184 first-void urine samples, 43 rectal swabs, 42 sperm samples, 32 urethral swabs, 30 pharyngeal swabs, 19 endometrial biopsy specimens, 12 Douglas/peritoneal fluid samples, eight nasopharyngeal aspiration samples, three intrauterine devices and 15 specimens of unknown origin.

Cervicovaginal specimens, self-collected vaginal swabs and sperm samples (100 μl or less, depending on the available volume) were collected either in Aptima vaginal swab transport medium (Hologic, Inc., USA) or in Universal Transport Medium (UTM, Copan, Italy) using FLOQSwabs (Copan, Italy), if a bacteriologic culture was also requested. In the latter case, 500 μl of UTM was transferred to an Aptima specimen transfer tube (Hologic Inc., USA). Urethral, rectal and pharyngeal swabs, crushed biopsy specimens, intrauterine devices, peritoneal fluids and nasopharyngeal aspiration samples (100 to 400 μl, depending on the available volume of sample) were collected in UTM and transferred in Aptima specimen transfer tube (Hologic Inc., USA) as described above. Urine samples were collected in Aptima urine specimen transport tubes. If urine was received in a sterile tube, 2 ml was immediately transferred into an Aptima urine specimen transport tube (Hologic Inc., USA).

### Aptima MG-TMA assay.

Specimens were collected in the appropriate Aptima sample collection medium and processed according to the manufacturer's instructions on the Panther system (Hologic Inc., USA). The MG-TMA CE-IVD assay, which is validated for urine and urogenital swabs and targets an 81-bp region of the 16S rRNA, was performed according to the manufacturer's instructions.

### In-house M. genitalium PCR.

Five microliters of the extraction and inhibition real-time PCR internal control (Dia-EIC/DNA; Diagenode, Belgium) was spiked into 200 μl of specimen that had been collected in Aptima medium according to the manufacturer's instructions. DNA from the 205-μl mixture was extracted using the MagNA Pure 96 DNA and Viral NA Small Volume kit (Roche Diagnostics, USA) on a MagNA Pure 96 instrument (Roche Diagnostics, USA). Extracted DNA was eluted in a final volume of 100 μl. The in-house PCR targeting the M. genitalium MgPa adhesin gene was performed on the cobas z 480 Analyzer (Roche Diagnostics, USA) as previously described (4, 6).

### RUO Alt1 and Alt2-TMA assays.

Two alternative M. genitalium detection assays, the RUO Aptima Alt1-TMA assay (8, 27, 28), targeting a different region of the M. genitalium 16S rRNA from the 16S rRNA region employed by the MG-TMA assay, and the Alt2-TMA assay, targeting a region of M. genitalium 23S rRNA different from the 23S RNA region targeted by the nested RT-PCR sequencing assay used below, were blindly performed on all 38 specimens with discrepant results and on 38 randomly selected MG-TMA- and in-house PCR-negative specimens. Both the Alt1- and Alt2-TMA assays have been validated to have an equivalent analytical sensitivity and specificity as the MG-TMA assay (8, 28) (D. Getman, personal communication). These tests were performed on the Panther system at Hologic, Inc., San Diego, CA, USA.

### Macrolide resistance detection.

All the confirmed M. genitalium-positive specimens were tested for macrolide resistance using three comparative assays. The two real-time PCR assays were performed on DNA extracts obtained from specimens in Aptima transport medium using the MagNA Pure 96 instrument (Roche Diagnostics, USA). The in-house 23S rRNA FRET PCR assay for the detection of macrolide resistance in M. genitalium-positive specimens, based on FRET technology coupled with melting curve analysis, was performed as previously described (17). The ResistancePlus MG assay (SpeeDx, Australia) was performed according to the manufacturer's instructions and used as previously described (20). This kit is based on the MNAzyme technology for the detection of M. genitalium (MgPa adhesin gene) and of the five most frequent 23S rRNA macrolide resistance-associated mutations (A2058G, A2059G, A2058C, A2059C, A2058T, Escherichia coli numbering). Finally, the nested RT-PCR sequencing assay was performed on specimens in Aptima transport media at Hologic Inc., San Diego, CA, USA, as previously described (11).

### Interpretation of results and statistical analyses.

The M. genitalium infection status of the specimens presenting discrepant results between the MG-TMA assay and the in-house PCR assay was determined using the Alt1- and Alt2-TMA assays by utilizing a consensus of two of the three assay results (not including the test assay being evaluated). Clinical sensitivity and specificity of the MG-TMA assay and of the in-house PCR were calculated by comparison to the M. genitalium status. Statistical comparisons were made using McNemar's test. The kappa statistic (κ) was used to evaluate the agreement between the MG-TMA assay and the in-house PCR results or the M. genitalium infection status. Statistical analysis was performed with the BiostaTGV website (marne.u707.jussieu.fr/biostatgv/). Differences with *P* values of <0.05 were considered significant.

Regarding the detection of M. genitalium macrolide resistance-associated mutations, the nested RT-PCR and Sanger sequencing assay was considered to be the reference gold standard for the detection of macrolide-resistance-associated mutations because the FDA considers PCR and sequencing to be the gold standard for target identification.

## Supplementary Material

Supplemental material
