# Supplementary material for: French Prospective Clinical Evaluation of the Aptima Mycoplasma genitalium CE-IVD Assay and Macrolide Resistance Detection Using Three Distinct Assays
Source: J Clin Microbiol. 2017 Oct 24;55(11):3194–200. doi: 10.1128/JCM.00579-17 (PMC5654902; doi:10.1128/JCM.00579-17)
Supplement: Supplemental material [file supp_55_11_3194__index.html]

Supplemental material 

# French Prospective Clinical Evaluation of the Aptima Mycoplasma genitalium CE-IVD Assay and Macrolide Resistance Detection Using Three Distinct Assays

## Supplemental material

- Supplemental file 1 -

  Table S1 (Results of *M. genitalium* detection on the 84 samples with at least one positive result and detection of macrolide resistance-associated mutations)

  XLSX, 1.6M
- Supplemental file 2 -

  Table S2 (Macrolide resistance results for the 72 specimens with an *M. genitalium*-positive infection status)

  PDF, 124K
