# Supplementary material for: French Prospective Clinical Evaluation of the Aptima Mycoplasma genitalium CE-IVD Assay and Macrolide Resistance Detection Using Three Distinct Assays
Source: J Clin Microbiol. 2017 Oct 24;55(11):3194–200. doi: 10.1128/JCM.00579-17 (PMC5654902; doi:10.1128/JCM.00579-17)
Supplement: Supplemental material [file JCM.00579-17_zjm999095676s2.pdf]

Table S1. Results of *M. genitalium* detection using the MG-TMA, in-house PCR, Alt1-TMA, and Alt2-TMa assays on the 84 samples with at least one positive result, and detection of macrolide resistance-associated mutations using nested RT-PCR sequencing, FRET PCR and ResistancePlus^{TM} MG assays.

See excel file attached.

Table S2. Macrolide resistance results of the 72 specimens with an *M. genitalium*-positive infection status using the nested RT-PCR sequencing assay as a reference, the ResistancePlus^{TM} MG assay and the in-house FRET PCR assays.

RT-PCR sequencing	ResistancePlus^{TM} MG	In-house FRET PCR	Number of specimens
WT	WT	WT	31
WT	WT	NA	7
WT	NA	WT	2
WT	NA	NA	24
WT	23S mutant	WT	2
A2059G	23S mutant	A2058/2059G	2
A2059C	23S mutant	A2059C	1
A2059G	23S mutant	NA	1
A2059C	WT	NA	1
A2059G	WT	NA	1

WT, Wild type ; NA, no amplification.
